# Melting Behavior of Direct Reduced Iron Pellets with Different Carbon Content in Molten Steel and Molten Slag

**DOI:** 10.3390/ma18204749

**Published:** 2025-10-16

**Authors:** Fabian Andres Calderon Hurtado, Joseph Govro, Arezoo Emdadi, Ronald J. O’Malley

**Affiliations:** Department of Materials Science and Engineering, Missouri University of Science and Technology, Rolla, MO 65409, USA; jgc3c@mst.edu (J.G.); emdadia@mst.edu (A.E.); omalleyr@mst.edu (R.J.O.)

**Keywords:** direct reduced iron (DRI), electric arc furnace (EAF), carbon, heat transfer coefficient, computational simulation

## Abstract

This study investigates the melting behavior of direct reduced iron (DRI) pellets in molten slag and steel baths, focusing on how the carbon content influences the melting rate through the stirring effects of gas evolution on heat transfer. A computational model using COMSOL Multiphysics 6.1 is developed to simulate the temperature profile at the pellet’s core and the gas evolution resulting from the reaction between FeO and carbon within the pellet. The model is validated using experimental data from this study as well as literature on the DRI pellet–molten slag system. Results indicate that, despite the increased enthalpy demand associated with the gas-generating reactions, higher carbon content enhances heat transfer within the pellet, leading to an increased melting rate. The computational model accurately predicts gas generation and temperature profiles, aligning well with experimental observations. Overall, the findings demonstrate that increasing the carbon content in DRI pellets accelerates the melting process.

## 1. Introduction

Due to rising environmental concerns and demands on the steelmaking industry to employ techniques that reduce greenhouse emissions, the industry is looking for different ways to produce low-emission steel [1,2,3,4,5]. The blast furnace–basic oxygen furnace (BF-BOF) route is responsible for approximately 70% of global steel production [6]. The production of one ton of steel via the BF-BOF steelmaking route generates approximately 1.8–2.2 tons of CO_2_ [7,8]. In contrast, steel production using an electric arc furnace (EAF) emits 102 kg of CO_2_ per ton of steel [9]. Therefore, there are opportunities in the use of EAFs compared to the BF-BOF route based on CO_2_ atmospheric emissions. This is aligned with steelmaking environmental goals to mitigate greenhouse emissions [7,8].

EAFs are well-suited for processing direct reduced iron (DRI) pellets or hot briquette iron (HBI) [10,11]. EAFs accounted for 28.6% of the world’s total crude steel production in 2023 (1892 million tons), and DRI production increased from 111.8 million tons in 2019 to 136.5 million tons in 2023 [6]. Although DRI production is a minor share of the total ferreous feed stock for global steel production, due to climate concerns, this technology may gain greater importance in the future, making this case a study of great interest [4,9,12].

Crucial metallic feedstocks for EAFs include steel scrap and virgin iron units, consisting of pig iron, HBI, and/or DRI. Although scrap is a viable material for steelmaking, controlling certain unrefinable impurities, such as copper and nickel, remains challenging due to their accumulation in recycled scrap [13]. This underscores the importance of using DRI in EAFs. Several researchers have conducted studies aimed at improving the efficiency of the DRI process. DRI pellets are produced in a vertical shaft furnace through a series of reactions with C or H_2_, as shown in Equations (1)–(6) [14].

(1) $$3{Fe}_{2}O_{3(s)}+{CO}_{\left(g\right)}\to 2{Fe}_{3}O_{4(s)}+{CO}_{2(g)}$$

(2) $${Fe}_{3}O_{4(s)}+{CO}_{\left(g\right)}\to 3{FeO}_{\left(s\right)}+{CO}_{2(g)}$$

(3) $${FeO}_{\left(s\right)}+{CO}_{\left(g\right)}\to {Fe}_{\left(s\right)}+{CO}_{2(g)}$$

(4) $$3{Fe}_{2}O_{3(s)}+H_{2(g)}\to 2{Fe}_{3}O_{4(s)}+H_{2}O_{\left(g\right)}$$

(5) $${Fe}_{3}O_{4(s)}+H_{2(g)}\to 3{FeO}_{\left(s\right)}+H_{2}O_{\left(g\right)}$$

(6) $${FeO}_{\left(s\right)}+H_{2(g)}\to {Fe}_{\left(s\right)}+H_{2}O_{\left(g\right)}$$

Although hydrogen direct reduced iron (H-DRI) pellets will contribute to the reduction in CO_2_ emissions in the iron and steel industry, it is still necessary to introduce some carbon into the EAF to create a foaming slag to improve the energy efficiency of the furnace to carburize the steel, control iron yield losses, and promote stirring to enhance the melting and refining processes [9,15,16,17].

Pfeiffer et al. [12] investigated H-DRI melting with hydrogen plasma smelting reduction (HPSR). This research showed that DRI with carbon melts faster than that without carbon. It was observed that the high stirring condition in the system is due to the electromagnetic stirring from the arc and the oxidation of carbon in the system, which reacts with the injected oxygen to produce CO gas. The article concludes that the reaction with carbon induces stirring, enhancing heat transfer and accelerating the melting rate.

In another separate study, Pfeiffer et al. [18] analyzed the melting behavior of DRI with different carbon content and HBI immersed in high- and low-carbon steel melts as well as high- and low-iron oxide slags. It was observed that carbon, whether present in the DRI sample or the melt, accelerated the dissolution process by reacting with residual iron oxide in the pellet or slag.

While Pfeiffer et al. [12] present interesting results focused on the melting behavior of DRI in HPSR, the conditions in this system can differ significantly from those in an EAF. As noted by Pfeiffer et al. [18], there is currently no computational simulation model capable of predicting the melting behavior under varying conditions, which limits a comprehensive understanding of the process.

Sharifi et al. [19] studied the melting dynamics of DRI pellets immersed in molten slag at temperatures varying from 1500 °C to 1600 °C. The research was focused on the effect of the initial carbon content and the preheating temperature of the DRI. The findings indicate that the carburization of DRI involves two stages. One reaction involves FeO within the pellet reacting with carbon, while the other occurs between FeO in the slag and the residual carbon in the pellet. The authors observed that carbon controls the two reaction stages, where high quantities of carbon increase the reaction rate. Ultimately, the preheating of the DRI pellet was found to enhance the decarburization rate during the first stage. Lin et al. [20] analyzed the melting of one and two H-DRI pellets with an 8 mm diameter in an EAF with molten steel using COMSOL Multiphysics. This study found that the H-DRI melting behavior can be divided into three stages: the formation of a solidified steel layer, the complete melting of the solidified steel layer, and the melting of the H-DRI body. They also found that the bonding among the pellets can be reduced by pre-heating the pellets or increasing the space between them.

The work of Sharifi et al. [19] offers a detailed result related to DRI’s experimental work in molten slag, describing how the carbon content affects the melting behavior process. On the other hand, Lin et al. [20] primarily focused on computational simulations of the H-DRI melting process. To the best of our knowledge, a comprehensive study that integrates both experimental work and computational modeling has not yet been fully developed—an area this research aims to address.

Kirschen et al. [21] examined the melting of DRI pellets and steel scrap for 16 industrial EAFs, focusing on the slag chemical composition. This study found that lowering the slag basicity from 2.0 to 1.4–1.7 reduces FeO losses in the slag and decreases the electrical energy required for melting by 8–17 kWh per ton.

Previous studies have analyzed the melting behavior of H-DRI pellets or DRI pellets with varying carbon content in molten slag or steel; however, there is no comparison of the effect of carbon content on the heat transfer and melting behavior of DRI. This paper investigates the impact of carbon on the melting behavior of DRI. Experimental data from DRI pellets immersed in molten slag and steel bath were utilized to validate the developed computational heat transfer model.

## 2. Heat Transfer and Gas Generation Model

### 2.1. Heat Transfer Model

To predict the heat transfer and temperature distribution, the conservation equation of energy is employed (Equation (7)), where ρ is the density, $C_{p}$ is the specific heat capacity at constant pressure, T is the temperature, u is the velocity, ∇.q is the conductive heat transfer term, and $Q^{*}$ contains other heat sources.

(7) $$\rho C_{p}\frac{\partial T}{\partial t}+\rho C_{p}u.\nabla T+\nabla .q=Q^{*}$$

In this equation, the conduction heat transfer is defined by Fourier’s law and shown in Equation (8):

(8) q=−k∇T

where q is the conductive heat flux, ∇T corresponds to the temperature gradient, and k is the thermal conductivity. The heat conductive term is coupled with the energy conservation equation. Heat conduction is considered within the pellet domain and the grown shell around the pellet, while heat convection around the pellet is attributed to the movement of the surrounding liquid bath. The heat conduction equation for a two-dimensional axisymmetric geometry in cylindrical coordinates is given in Equation (9). The convective heat transfer was considered according to Equation (10).

(9) $$q_{conduction}=k\left(\frac{1}{r}\frac{\partial }{\partial r}\left(r\frac{\partial T}{\partial r}\right)+\frac{\partial }{\partial z}\left(\frac{\partial T}{\partial z}\right)\right)=\rho C_{p}\frac{\partial T}{\partial t}\;\;\;\;\;\;\;\;\;\;\;\;\;0<r<R\;\mathrm{or}\;r<R_{shell},\;\mathrm{where}\;R\;\mathrm{is}\;\mathrm{the}\;\mathrm{radius}\;\mathrm{of}\;\mathrm{the}\;\mathrm{pellet}$$

(10) $$q_{convection}=h(T_{\infty }-T_{s})\;\;\;r=\mathrm{Rshell}$$

In the previous equations, h is the heat transfer coefficient, $T_{\infty }$ is the temperature in the molten slag or steel bath, and $T_{s}$ is the temperature at the surface of the pellet. We assumed that h is constant for the DRI pellets submerged in a steel bath. For the DRI pellets’ melting behavior in molten slag, h varied with the gas evolution rate according to Equation (11) [22]. As heat convection is related to the movement of the liquid bath around the pellet, which is primarily caused by the gas evolution of CO, it is vital to establish a relationship between the gas evolution rate (g), resulting from the reduction of iron oxide with carbon, and the heat convection through the heat transfer coefficient.

(11) $$h=8.6\times 10^{-3}g^{1/4}$$

The latent heat of fusion determines the heat needed for the solid/liquid phase transformation, while the balance between conductive and convective heat fluxes influences the rate of phase change. COMSOL Multiphysics 6.1 utilizes the concept of apparent heat capacity to account for phase transformations. The apparent heat capacity method describes the phase change of the material by modifying the specific heat capacity ($C_{p}^{\prime }$) and considering the latent heat of fusion. More details can be found in [14].

Two possible scenarios can occur at the interface of the pellet and the steel bath/molten slag:

(1) If the pellet’s surface temperature is below its melting point, there are no transformations, and the heat transfer is defined by Equation (12):

(12) $$q_{convection}=h\left(T_{\infty }-T_{s}\right)$$

(2) Once the pellet’s surface reaches its melting point, the heat required for the solid-to-liquid phase transformation is incorporated into Equation (12) in terms of the apparent heat capacity shown in Equation (13):

(13) $$q_{conduction}=\rho \frac{\partial (C_{p}^{\prime }T)}{\partial t}+h\left(T_{\infty }-T_{s}\right)$$

**Figure 1:**
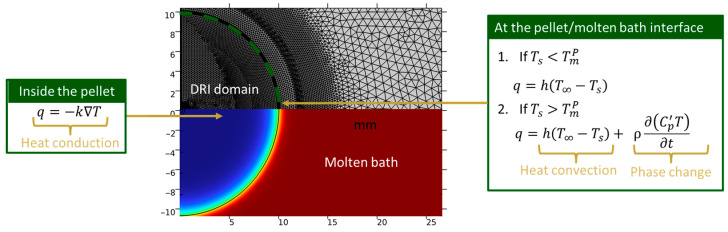
Schematic of the computational domain showing regions where heat conduction and convection are applied. DRI domain (blue color), and molten bath domain (red color).

Figure 1 illustrates the domain in which heat conduction and convection are applied.

### 2.2. Gas Generation Model

In this study, the carbon content and iron oxide were defined within the context of the DRI pellet system. As previously mentioned, gas generation is used to determine the heat transfer coefficient. Specifically, the generation of CO gas from the reduction of FeO by carbon is utilized to analyze heat transfer within the system of DRI pellets immersed in molten slag, as discussed later in this research.

For the gas generation model, the reduction of iron oxide with carbon was investigated using Equation (14). This reaction follows first-order kinetics with respect to FeO, and its rate (*r*) is given by Equation (15). A simplified rate expression is used in Equation (15), considering only iron oxide, as FeO is the limiting reactant in these cases.

(14) FeO+C=Fe+CO

(15) r=k[FeO]

where [FeO] is the concentration of iron oxide and k is the rate constant defined through the Arrhenius equation shown in Equation (16):

(16) $$k=Ae^{\left(\frac{-Q}{RT}\right)}$$

In this equation, A is the pre-exponential factor, Q is the activation energy of the reaction of FeO with C, and R is the gas constant. From the reaction rate, we can estimate the quantity of moles of CO generated per unit time and volume. Therefore, a volume integration of the reaction rate over the pellet will show the moles of CO produced per unit time, and using the gas equation below (Equation (17)), it is possible to determine the CO gas generation. While numerous equations are available in the literature for calculating gas generation, the complexity and computational demands of the simulation model make it more practical to use simplified models that yield results consistent with experimental observations.

(17) PV=nRT

where P is pressure, V is volume, n is moles, R is the gas constant, and T is temperature. This equation can be utilized to calculate the volume of gas generated per unit time.

### 2.3. Porosity-Dependent Thermophysical Properties

The DRI pellet porosity was estimated according to Archimedes’ method according to ASTM C20 [23]. The measured open porosity was 0.54 ± 0.02 for the DRI pellets with 1.3 wt. % carbon. Our previous study showed that the difference between the open porosity and the total porosity (open + closed porosity) is negligible [14].

Density, ρ, specific heat capacity, $C_{p}$, and thermal conductivity, *k*, are corrected using Equations (18)–(20) [13,24,25].

(18) $$\rho_{pellet}=\rho_{s}(1-e)$$

(19) $$C_{p}=\frac{\left(C_{p,f}V_{f}+C_{p,m}V_{m}\right)}{V_{f}+V_{m}}$$

(20) $$k_{eff}=k_{m}(\frac{1-e}{1+ne^{2}})$$

where $\rho_{s}$ is the density of the solid phase, e is the porosity of the pellet, $C_{p,f}$ represents the specific heat capacity of the air inside the pores, $V_{f}$ denotes the volume of the pores, $C_{p,m}$ shows the specific heat capacity of the pellet, $V_{m}$ represents the volume of the pellet, $k_{m}$ is the thermal conductivity of the pellets, and n is a constant equal to 11 for DRI pellets [22]. 

### 2.4. Boundary Conditions

**Figure 2:**
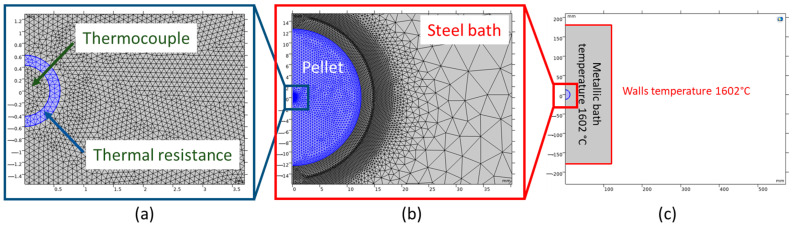
Geometry design of the DRI pellet: (**a**) Thermal resistance between the thermocouple and pellet, with arrows indicating the thermocouple and thermal resistance. (**b**) The embedded pellet (blue color) is located in the molten bath, surrounded by a refined meshing structure at the interface. (**c**) Geometry and initial temperature conditions [14]. Images imported from COMSOL Multiphysics 6.1 software.

The computational domain and mesh detail for simulating a DRI pellet immersed in a molten bath are shown in Figure 2. In all simulations, thermal resistance between the thermocouple and the DRI pellet was accounted for, arising from the high-temperature adhesive used for bonding, which possesses a thermal conductivity of 0.04 [W m^−1^ K^−1^] [26]. The crucible walls, with dimensions of 240 mm in diameter and 360 mm in height, were maintained at a constant temperature of 1602 °C. Convective heat transfer between the steel bath/molten slag and the DRI pellet was modeled based on the heat transfer framework in Section 2.1. The initial temperatures were 77.3 °C for the DRI pellet and 1602 °C for the steel bath/molten slag. The computational domains—including the thermocouple, thermal resistance, DRI pellet, and molten bath—were meshed using free triangular elements with linear Lagrangian shape functions. The mesh sizes were specified as follows: 0.03 mm near the melting interface (between the DRI pellet and the steel bath/molten slag), 0.05 mm in the thermocouple and thermal resistance regions, and 3 mm for the remaining domain. A mesh quality study revealed an average mesh element quality of 0.937, utilizing approximately 5,300,000 elements for the DRI in the molten bath simulation. The mesh quality metric, with values above 0.9 indicating high-quality meshing, validated the suitability of the mesh for an accurate simulation [27].

## 3. Experimental Setup

For the experiments carried out in this study, an alumina crucible with a capacity of 90 kg and a furnace supplied by Inductotherm Corp., Rancocas, NJ, USA, were employed. The dimensions for the crucible are 360 mm in height and 240 mm in diameter. Clevland Cliffs, Corpus Christi, TX, USA provided DRI pellets with 1.3 wt. % carbon. The mass proportion of the DRI samples is shown in Table 1.

A pneumatic frame was used to immerse the DRI samples 18 cm in molten steel, whose chemical composition is detailed in Table 2. For the computational simulation, the thermal properties-thermal conductivity, specific heat capacity, and density were calculated based solely on the iron and carbon weight percentages in the pellets. The experimental design for the DRI pellet submersion time is outlined in Table 3.

**Figure 3:**
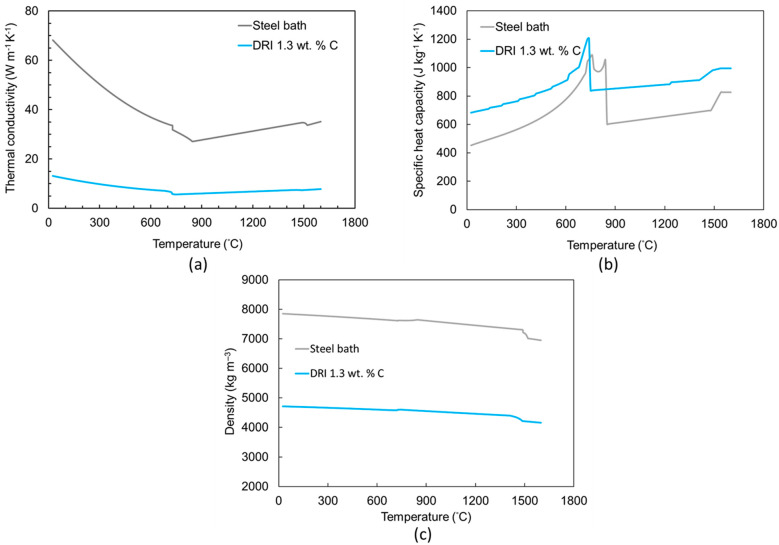
Calculated (**a**) thermal conductivity, (**b**) specific heat capacity, and (**c**) density of the DRI pellets and steel bath at various temperatures using JmatPro v14.

It is important to note that some samples fell into the steel bath, rendering their results unrecoverable. To prevent inductive stirring in the steel bath, the furnace was switched off during pellet submersion. The thermal physical properties were determined using JmatPro-v14 through the general steel section and thermo-physical properties and by defining the chemical composition. The information is presented in Figure 3 and Table 1, Table 2, Table 3, Table 4 and Table 5.

**Figure 4:**
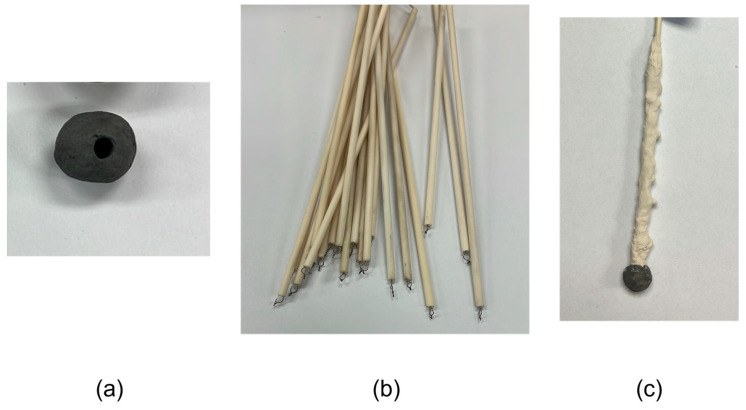
Experimental setup: (**a**) Drilled DRI pellet, (**b**) thermocouple and alumina tube, and (**c**) attached thermocouple to the DRI pellet, coated with zirconia.

Five samples were weighed, and their diameters were measured before the experiments. A milling machine equipped with titanium-coated 1/8-inch drill bits was used to drill the samples to their center. K- and S-type thermocouples were utilized in the experiment. Alumina–silicate cement was used to secure the thermocouple to the sample. To avoid fractures due to thermal shock after immersion, a zircon coating was applied to the alumina tube housing the thermocouple. The configuration is shown in Figure 4.

**Figure 5:**
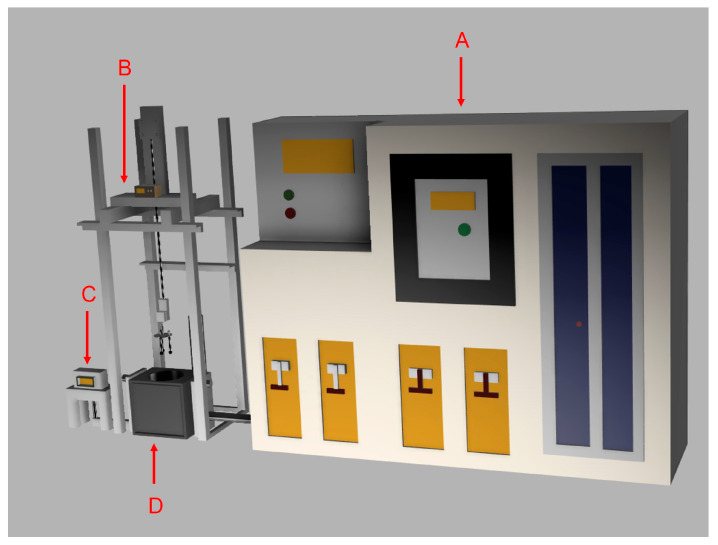
General setup: (A) power generation control device, (B) sample mounting stage, (C) pneumatic control device, and (D) induction furnace.

**Figure 6:**
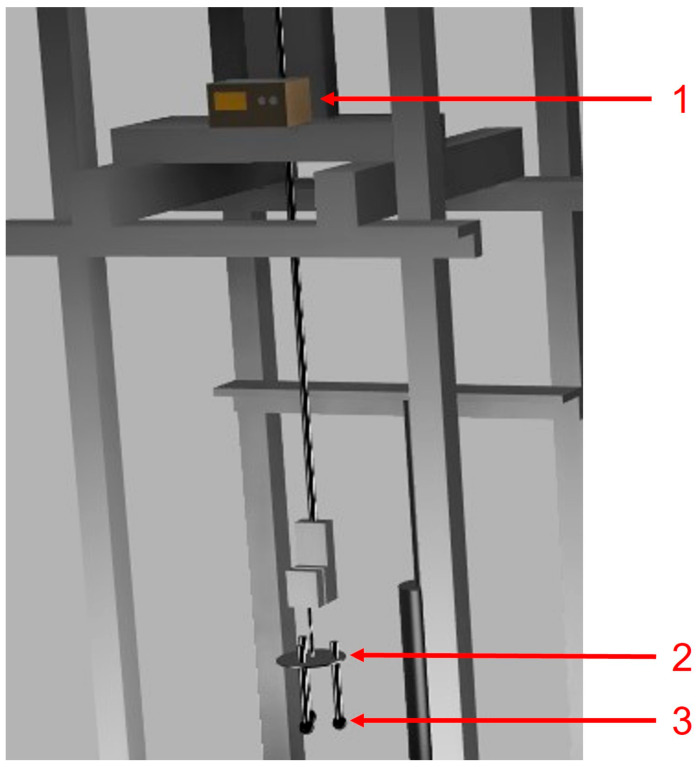
Sample setup: (1) GL220 midi data logger, (2) metallic fixture, and (3) samples of DRI pellets.

The samples immersed in molten steel were secured in four identical fixtures, each designed to hold three samples at a depth of 18 cm within the molten bath. The alumina tubes were affixed to the steel fixtures using cement. The molten steel temperature was maintained at 1602 °C and continuously monitored with a temperature probe from Heraeus Electro-Nite, Hartland, WI, USA. The temperature at the center of the samples was recorded using a GL220 midi data logger from Graphtec Corporation, Irvine, CA, USA, while the thermocouples were supplied by Omega, Norwalk, CT, USA. The setup is illustrated in Figure 5 and Figure 6.

## 4. Results and Discussion

### 4.1. Temperature Profiles

**Figure 7:**
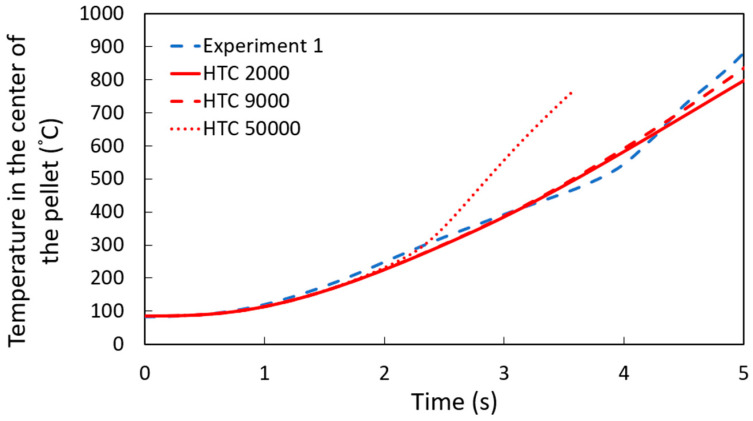
Temperature profile at the center of a 1.3 wt. % C DRI pellet with different heat transfer coefficients W m^−2^ K^−1^.

The simulations assumed a constant heat transfer coefficient to study DRI pellets immersed in a steel bath. Various values of the heat transfer coefficient were tested to determine the one that best aligned with the experimental data. As it is shown in Figure 7, the simulation results for a pellet containing 1.3 wt. % C, with a heat transfer coefficient of 9000 W m^−2^ K^−1^, showed the best agreement with the experimental measurement, which matches the results of the shell thickness measurements shown later.

**Figure 8:**
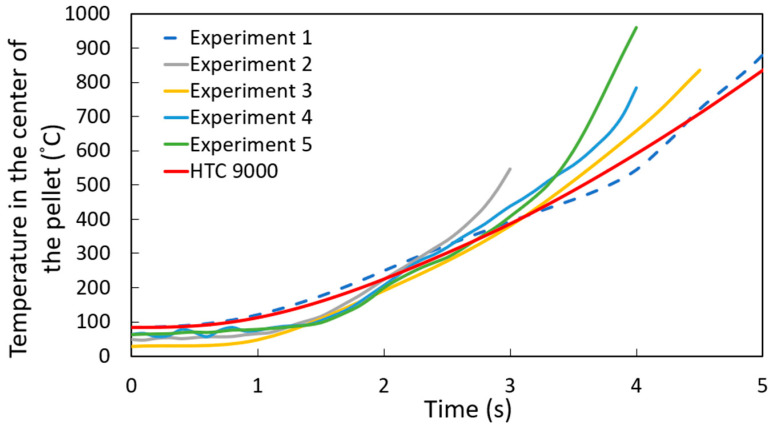
Temperature profile at the center of the DRI 1.3 wt. % C pellet with a heat transfer coefficient of 9000 W m^−2^ K^−1^.

Figure 8 shows the experimental results for the temperature profile at the center of the DRI pellet—a ten-point-five-millimeter-diameter pellet with 1.3 wt. % C—immersed in a steel bath; the red line represents the computational simulation result.

**Figure 9:**
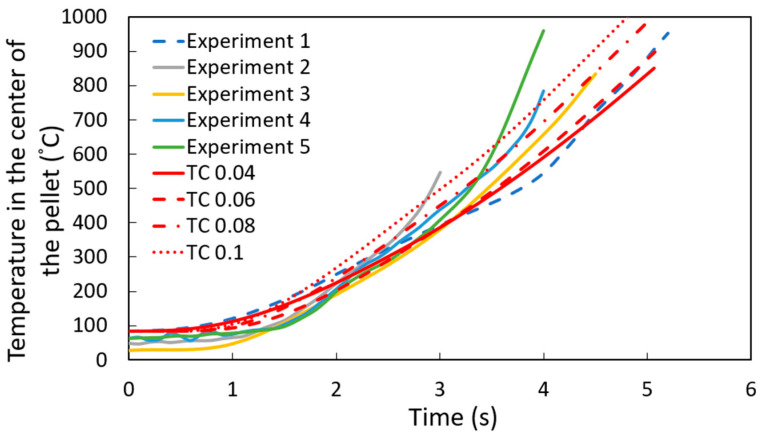
Temperature profile at the center of the DRI 1.3 wt. % C pellet with a heat transfer coefficient of 9000 W m^−2^ K^−1^, varying the thermal conductivity (TC) of the cement with units W m^−1^ K^−1^.

As shown in Figure 8, the computational simulation model accurately captures the temperature profile at the center of the pellet for experiment 1. However, there is some dispersion in the results for experiments 2 through 5. This variation can be explained by differences in the high-temperature adhesive used to attach the thermocouples to the DRI pellets, as the adhesive type varied between experiments. Therefore, the thermal conductivity of the high-temperature adhesive was varied between 0.04 and 0.1 W m^−1^ K^−1^ (the reported range of thermal conductivity for these high-temperature adhesives) [26]. Figure 9 corroborates that this variation in thermal conductivity can account for the dispersion observed in experiments 2 to 5.

**Figure 10:**
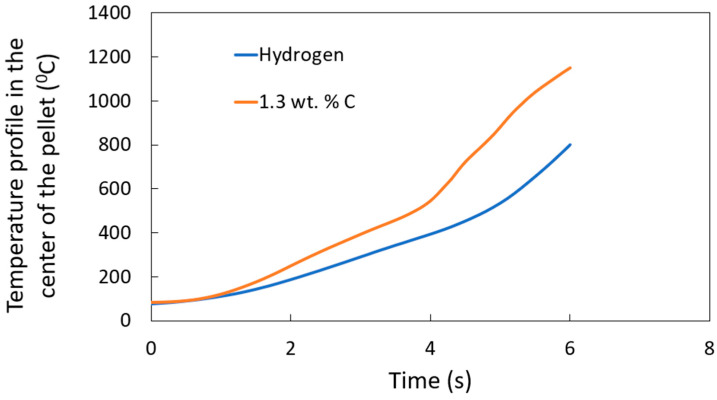
Temperature profile at the center of the sample for different carbon contents: H-DRI (0 wt. % carbon) and DRI (1.3 wt. % carbon).

The temperature profile for H-DRI [14] and DRI with 1.3 wt. % carbon is compared in Figure 10. As expected, DRI pellets with higher carbon content exhibit higher heating rates than those with zero carbon content. This can be explained by the stirring around the pellet caused by the CO gas generation from the reaction between the carbon and the iron oxide. A condensed equation for the process of FeO reduction with carbon is presented in Equation (19).

The DRI pellet with 1.3 wt. % carbon shows significant CO gas generation. This enhances stirring around the pellet, improving heat transfer and accelerating the melting process. In contrast, H-DRI pellets contain no carbon, resulting in no CO gas generation and no enhancement in heat transfer to the pellet.

**Figure 11:**
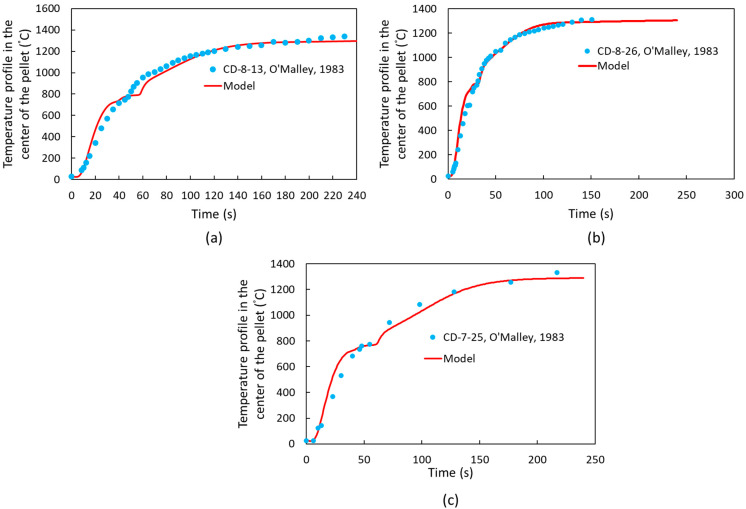
Temperature profile at the center of DRI pellets based on heat transfer coefficient dependent on the CO evolution rate (Equation (11)) for (**a**) Sample CD-8-13 [22], (**b**) Sample CD-8-26 [22], and (**c**) Sample CD-7-25 [22].

Following model validation, additional cases from the literature, specifically the studies by R. J. O’Malley [22], were examined. The characteristics of the pellets, including variations in pellet diameter, mass, and carbon content, are presented in Table 6, while the thermophysical properties of the slag are detailed in Table 7 [22]. Other thermophysical properties can be found in [22]. The temperature profiles at the center of the pellets are presented in Figure 11. Based on these profiles, the computational simulation model accurately predicts temperature variations in DRI pellets with varying carbon content and geometry.

### 4.2. Shell Thickness and Gas Generation

**Figure 12:**
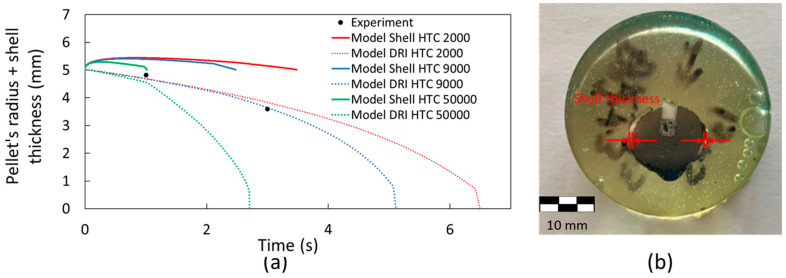
Shell thickness measurements in a steel bath for a DRI pellet with 1.3 wt. % C and a heat transfer coefficient varying between 2000 and 50,000 W m^−2^ K^−1^. (**a**) Shell thickness behavior (continuous line), DRI melting behavior (discontinuous line), and experimental results (black points). (**b**) Cross-section of a DRI pellet with 1.3 wt. % C mounted on epoxy resin.

**Figure 13:**
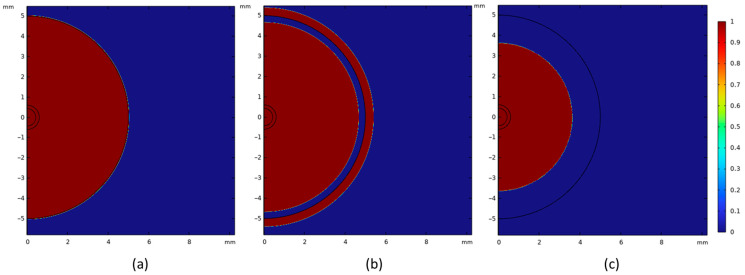
Shell thickness behavior of a DRI pellet with 1.3 wt. % C in a steel bath with a heat transfer coefficient 9000 W m^−2^ K^−1^. (**a**) Time 0 s. (**b**) Time 1 s. (**c**) Time 3 s.

Figure 12a shows the shell thickness for the DRI pellets with 1.3 wt. % carbon. For this case, it is observed that a heat transfer coefficient of 9000 W m^−2^ K^−1^ in the model provides a better prediction of the melting behavior of the DRI pellet compared to the experimental results. Five samples were used to analyze shell thickness; however, only two were successfully recovered for measurement. An example of the shell thickness measurement is shown in Figure 12b. The experimental results for the pellet’s radius and shell thickness, as presented in Figure 12a, align well with the computational simulation, confirming the model’s ability to accurately predict the melting behavior of a DRI pellet containing 1.3 wt. % carbon. As previously shown, the melting temperatures of the steel bath and the DRI with 1.3 wt. % C differs due to the carbon content (1794 K and 1761 K, respectively). Therefore, as a shell of steel forms around the pellet, the pellet begins to melt before the shell has fully melted. This can be observed in Figure 13: At time 0 s, the DRI pellet is surrounded by the steel bath without any shell thickness (Figure 13a); when the shell starts to melt, the DRI has already begun melting (Figure 13b); and finally, the shell is fully melted in Figure 13c.

### 4.3. DRI Pellet Gas Generation

**Figure 14:**
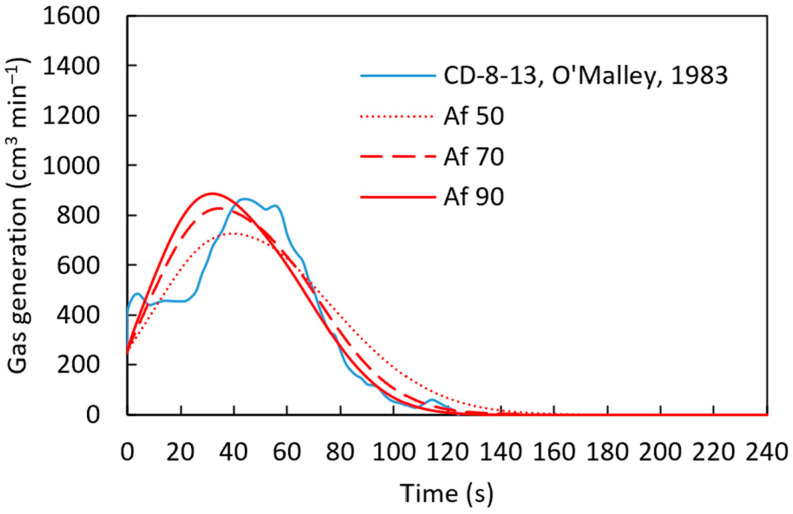
Gas generation for sample CD-8-13 [22] with varying pre-exponential factor and an activation energy of 83,680 J mol^−1^ using a heat transfer coefficient defined by $h=8.6\times 10^{-3}\;g^{1/4}$.

For the gas generation studies, we employed the experimental data from [22], as summarized in Table 6. For this case, the activation energy required in the last stages of the reduction of iron oxide is considered to be $Q=83,680\;J\;{\mathrm{mol}}^{-1}$ [22]. Then, the pre-exponential factor was adjusted ($Af=50,\;\;70,\;\mathrm{and}\;90\;s^{-1}$) to achieve agreement between the experimental and computational simulation results for the sample CD-8-13, as shown in Figure 14.

**Figure 15:**
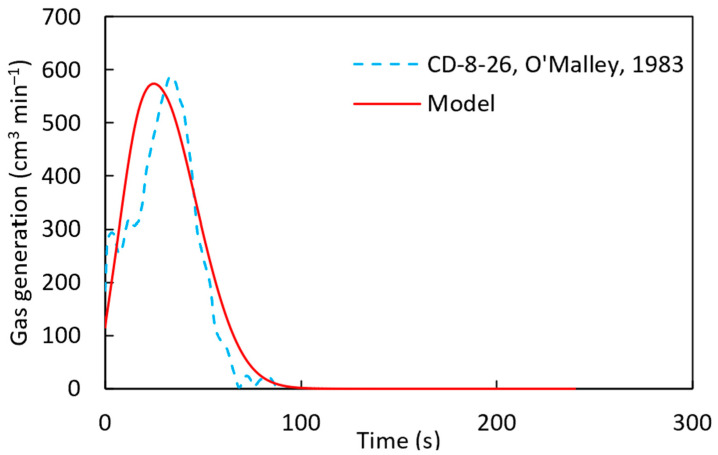
Gas generation for sample CD-8-26 [22] of the DRI pellet with a pre-exponential factor of 90 s^−1^ and the activation energy of 83,680 J mol^−1^ using a heat transfer coefficient defined by $h=8.6\times 10^{-3}\;g^{1/4}$.

According to Figure 14, with a pre-exponential factor of $90\;s^{-1}$ and the activation energy of $83,680\;J\;{\mathrm{mol}}^{-1}$, established from the literature [22], the computational simulation results are in close agreement with the experimental results. The calculated gas generation rate is compared with the experimental measurement in Figure 15. At the beginning of the simulation, discrepancies were observed between the experimental results and the computational predictions. This can be attributed to localized variations in carbon content within the pellet, which affect gas generation rates.

**Figure 16:**
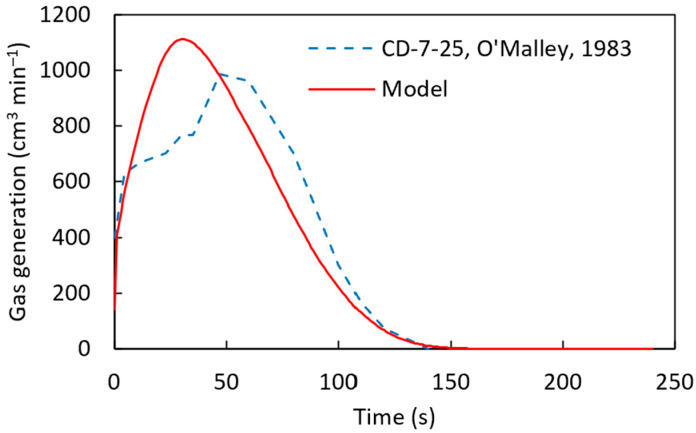
Gas generation for the CD-7-25 [22] sample with a pre-exponential factor of 90 s^−1^, an activation energy of 83,680 J mol^−1^ using a heat transfer coefficient defined by $h=8.6\times 10^{-3}\;g^{1/4}$.

From Figure 14 and Figure 15, it can be concluded that the computational simulation model accurately predicts the experimental gas generation results. With this validation, the model was applied to the CD-7-25 sample, as shown in Figure 16. However, some discrepancies were observed between the computational and experimental results, similar to Figure 15. This difference can be attributed to the model’s assumption of a uniform distribution of carbon and iron oxide throughout the pellet, whereas, in reality, localized variations in carbon and FeO concentrations may exist. Such variations can significantly influence the melting behavior of DRI pellets, as discussed in the work of Hesham Ahmed [29].

## 5. Conclusions

This study successfully investigated the melting behavior of direct-reduced iron (DRI) pellets with varying carbon content in molten steel and slag, utilizing a combination of computational modeling and experimental validation. The developed computational model accurately predicted the temperature profiles, gas evolution, and melting behavior of DRI pellets, demonstrating strong agreement with experimental data.

Key findings indicate that the presence of carbon in DRI pellets significantly enhances heat transfer, accelerates melting, and promotes stirring due to the evolution of CO gas. Higher carbon content corresponded to increased heat transfer and faster melting rates, with computational simulations effectively capturing these trends. Additionally, the model’s ability to predict gas generation was validated using literature data, further supporting its reliability in simulating DRI pellet melting behavior.

These results underscore the significance of carbon content in enhancing DRI melting efficiency, particularly in electric arc furnace (EAF) steelmaking. The findings provide valuable insights for improving process efficiency and energy utilization in steel production. Future work could investigate the impact of non-uniform carbon distribution within DRI pellets and its influence on localized melting behavior, thereby refining the model further.

## Figures and Tables

**Table 1:** Chemical composition of the DRI samples.

Sample (wt. %)	C	P	SiO_2_	CaO	Al_2_O_3_	MgO	Fe	Metallization (%)
1	1.3	0.04	3.37	0.90	0.38	0.63	90.2	96.6

**Table 2:** Chemical composition of the steel bath.

Sample (wt. %)	C	Mn	Si	Fe
Steel bath	0.190	0.020	0.140	99.650

**Table 3:** Experimental design of the immersion time for DRI pellets with different carbon content.

DRI 1.3 wt. % C	
Sample	Time (s)
1	1
2	2
3	3
4	4
5	7

**Table 4:** Thermophysical properties of the steel bath.

Parameters	Symbol	Steel Bath
Bath temperature [K]	$T_{\infty }$	1875 *
Melting temperature [K]	$T_{m}$	1794 [28]
Pellet diameter [m]	D	0.0105 *
Latent heat of fusion [kJ kg^−1^]	$L_{f}$	270 [28]

* Based on experimental measurement.

**Table 5:** Thermophysical properties of DRI pellets.

Parameters	Symbol	DRI 1.3 wt. % C
Initial temperature [K]	T	357 *
Melting point [K]	$T_{m}$	1761 [28]
Latent heat of fusion [kJ kg^−1^]	$L_{f}$	302 [28]

* Based on experimental measurement.

**Table 6:** Diameter, mass, and chemical composition of DRI pellets.

Sample [22]	Diameter (mm)	Mass (g)	Chemical Composition (wt. %)			
			Carbon	Gangue	FeO	Fe
CD-8-13	24.9	32.0	1.98	5.74	9.30	82.98
CD-8-26	19.2	14.8				
CD-7-25	25.0	32.0	2.37	6.75	14.64	76.24

**Table 7:** Thermophysical properties of the DRI pellets.

Parameters	Samples [22]			
	CD-8-13	CD-8-26	CD-7-25	Slag
Density [kg m^−3^]	3960		3880	2900
Initial temperature [°C]	25		25	1400
Melting temperature [°C]	1503		1537	1316
Thermal conductivity [W m^−1^ K^−1^]	2.5		2.5	1.17 (solid) 1.33 (Liquid)
Latent heat of fusion [kJ kg^−1^]	293		247	920

## Data Availability

The original contributions presented in the study are included in the article; further inquiries can be directed to the corresponding author.
